# Predicting students’ continued intention to use E-learning platform for college English study: the mediating effect of E-satisfaction and habit

**DOI:** 10.3389/fpsyg.2023.1182980

**Published:** 2023-06-23

**Authors:** Ping Deng, Bing Chen, Li Wang

**Affiliations:** ^1^Basic teaching department, Hezhou University, Hezhou, China; ^2^School of Foreign Languages for Business, Guangxi University of Finance and Economics, Nanning, China; ^3^School of Foreign Languages, Hunan International Economics University, Changsha, China

**Keywords:** UTAUT2, e-learning platform, learning value, e-satisfaction, habit

## Abstract

Using technology in education facilitates knowledge dissemination expediently while broadening and deepening learning modes and content diversity. As an information technological innovation, E-learning platform is widely used to learn college English. However, few studies have explored the motivations for students’ e-satisfaction and continued intention towards using it for college English study. Based on the extended Unified Theory of Acceptance and Use of Technology (UTAUT2), this study identifies the influencing factors for the continued usage intention and tests the mediating role of e-satisfaction and habit. Six hundred and twenty-six usable responses from Guangxi were analyzed with partial least squares structural equation modelling. Results show that performance expectancy, learning value, hedonic motivation and habit positively affects students’ continued usage intention, e-satisfaction positively mediates the relationship between the antecedents and continued usage intention, and habit positively mediates the relationship between e-satisfaction and continued usage intention. The research provides guidelines for the successful implementation of e-learning platform for college English study and key references for improvement of students’ engagement and satisfaction experience with using e-learning platform for college English study.

## Introduction

The application of information technology in education has greatly promoted the development of higher education. In particular, it has led to the creation of many efficient and economic information technological tools that are designed to facilitate learning and deliver knowledge without time and place restrictions. E-learning platform (ELP) is such an innovation driven primarily by information technology. ELP provides a versatile and economic method to learn and share knowledge with the help of electronic devices and an internet connection at any time and place (Osei et al., 2022). ELP offers asynchronous and synchronous communication channels for students to undertake online learning according to their own learning time schedule, and it plays a great role in enhancing learning performance in higher education (Ain et al., 2016; Prasetyo et al., 2021). ELP has even replaced traditional face-to-face teaching during the COVID-19 pandemic (He, 2020; Zou et al., 2021), providing a new way for students to learn college English, which is a vital part of Chinese higher education to develop students’ abilities, knowledge, and overall quality.

The use of ELP has revolutionized education systems and changed learning styles all over the world (Raman and Thannimalai, 2021). As a result, educational institutions have invested heavily in accelerating and supporting educational informationization (Ain et al., 2016). However, despite the investment and effort, the results have not always been as beneficial and desirable as expected. In some cases, students have reported low satisfaction, low participation, and low learning efficiency when using ELP (Cai et al., 2020; Li et al., 2020; Qiao et al., 2021; Xu et al., 2022). Ultimately, the success of ELP depends on whether students are satisfied with their experience of using it and are willing to continue using it in future studies (Ismail et al., 2016). This highlights the importance of initial acceptance and continued intention to use the technology in the long term (Bhattacherjee et al., 2008).

While many studies have examined factors influencing university students’ initial adoption of ELP (Raman and Thannimalai, 2021; Tandon et al., 2021; Xu et al., 2022; Zacharis and Nikolopoulou, 2022), few have explored the factors contributing to their continued use, particularly in college English study. In order to address this gap in the literature, the present study aims to delve deeper to identify the factors that influence students’ continued use of ELP for college English study. Moreover, while previous research has investigated the impact of antecedents on customers’ satisfaction and adoption of new technologies (Alalwan, 2020; Santosa et al., 2021; Siyal et al., 2021), few have examined the mediating role of e-satisfaction and habit in the relationship between antecedents and continued use in college English study (Rai, 2020). Therefore, this study will also explore the mediating effect of e-satisfaction and habit in the relationship between predictors and continued usage intention. By identifying the key factors that affect students’ continued intention to use ELP and the mediating role of e-satisfaction and habit, this study will provide a more comprehensive understanding of the factors that would increase the successful implementation of ELP and contribute to students’ sustained use of ELP for college English study. The findings of this research will offer practical implications for educators, policymakers, and ELP developers, who can better understand the factors and design effective strategies to promote the continued use of ELP for college English learning.

## Theoretical background and hypothesis development

### Theoretical background

The Unified Theory of Technology Acceptance and Use of Technology (UTAUT) is a widely used model that was proposed to explain and predict the use of new technologies (Venkatesh et al., 2003). The original UTAUT model included four constructs: performance expectancy, effort expectancy, social influence, and facilitating conditions. However, research later found that three additional constructs could improve the model’s predictive and explanatory power: hedonic motivation, price value, and habit (Venkatesh et al., 2012). As a result, the UTAUT2 model was created, which can now explain over 70% of the variance for the intention to adopt and use new technologies (Venkatesh et al., 2012).

In the educational context, a large amount of studies have used this model to investigate the effect of antecedents on students’ and teachers’ intention to adopt and use new technologies (Cacciamani et al., 2018; Dajani and Abu Hegleh, 2019; Nikolopoulou et al., 2020; Raman and Thannimalai, 2021; Zacharis and Nikolopoulou, 2022). However, few studies have investigated students’ continued usage intention. Continued usage intention refers to the user’s willingness to continue using a new technology over an extended period of time, even after the initial adoption and use phase (Santosa et al., 2021; Abbasi et al., 2022; Gao, 2023; Maduku and Thusi, 2023). In the context of UTAUT2, continued intention is measured as the intention to continue using a technology for a specific purpose, such as using an ELP for college English study. Understanding the factors that affect continued intention is important as it can impact the long-term success and sustainability of the technology. This research seeks to bridge this gap by adopting the UTAUT2 model to study the factors that impact students’ continued use of ELP for college English study.

In order to more thoroughly examine and validate the potency of UTAUT2, Venkatesh et al. (2012) emphasized the importance of incorporating constructs that can reflect the very nature of the targeted subject in specific contexts. In an effort to better investigate the factors that impact students’ continued usage intention, the concept of price value is replaced with that of learning value, as students do not need to pay to benefit from ELP; rather, they invest their time and energy to study and obtain skills and knowledge through ELP (Ain et al., 2016). Previous research has shown that learning value has a significant and positive influence on students’ intention to use new technology in the learning context (Zacharis and Nikolopoulou, 2022). Furthermore, given that few studies have explored the mediation role of students’ e-satisfaction for the relationship between antecedents and continued usage intention and the mediating effect of habit on the relationship between e-satisfaction and continued usage intention, this study attempts to fill this gap by incorporating learning value into the extended UTAUT2 model and investigates the mediating effect of e-satisfaction and habit to better understand the relationship between the antecedents and continued willingness to use ELP for college English study. By doing so, a more comprehensive and nuanced understanding of the factors that affect students’ usage intention of ELP can be obtained.

### Hypotheses development

*Performance expectancy (PE)* refers to the perception of how much a new technology can improve a user’s performance or how beneficial it is in completing certain activities (Venkatesh et al., 2012). Research has shown that PE strongly influences a user’s willingness to continue using new technologies (Santosa et al., 2021; Sasongko et al., 2022; Wu et al., 2022). Users who perceive the benefits of a new technology are more likely to continue using it over time. In the learning context, it is assumed that students are more willing to continuously use ELP if they believe that it is useful and helpful in performing various English study activities, and it enables them to achieve desirable outcomes more efficiently. Therefore, this research posits that:

*H1*: PE positively impact students’ continued intention to use ELP for college English study.

*Effort expectancy (EE)* refers to individuals’ beliefs about the effort or ease of using a particular new technology, or their perception of how easy or difficult it is to use a new technology (Venkatesh et al., 2003). This concept is important because it influences whether users will continue to use the technology in the future. Research has shown that EE has a positive impact on users’ willingness to use a new technology in the future (Yan et al., 2021; Abbasi et al., 2022). This study examines whether students perceive the operation instructions of ELP as easy to understand and clear, and whether they find it convenient and effortless to use when completing English tasks and activities. If students feel that the technology is easy to use and does not require much mental or physical effort, they are more likely to continue using it in their future college English studies. Therefore, the following hypothesis can be proposed:

*H2*: EE positively impacts students’ continued intention to use ELP for college English study.

*Social influence (SI)* refers to the impact of the external environment, or others’ beliefs, on an individual’s willingness to use a particular new technology (Venkatesh et al., 2003). The influence mainly comes from the social pressure of the external environment surrounding the individual. Several studies have shown the impact of SI on individuals’ decision and continued intention to use new technologies. For instance, Gao (2023) found that SI is a crucial factor that affects individuals’ decision to adopt new technologies. Similarly, Venkatesh et al. (2003, 2012) and Zacharis and Nikolopoulou (2022) have also found that SI plays an vital role in individuals’ intention to use new technologies. In this research, SI mainly refers to the influence of peers, family, friends and teachers’ ideas on the use of ELP. If external factors and beliefs are positive regarding students’ intention to use ELP for college English study, then their continued usage intention would be enhanced. The following hypothesis investigates this relationship:

*H3*: SI positively impacts students’ continued intention to use ELP for college English study.

*Facilitating conditions (FC)* refer to the availability of technical support and resources provided by the organization to support the use of a new technology (Venkatesh et al., 2003). This can include things like assistance from IT personnel, access to necessary software and hardware, and a stable internet connection. Without these resources, users may be hindered from using a new technology on a continuous basis (Nanayakkara, 2007). However, if FC are present, users are inclined to continue using the technology (Tandon et al., 2021; Gao, 2023). In this research, FC specifically refers to the accessibility of technical support, tools, facilities, stable internet connection, and other compatible technologies that support the use of ELP for college English study. If students have access to these resources and technical support, as well as other forms of support from their universities, they will be more motivated to continue using ELP for college English study. Therefore, the following hypothesis can be posited:

*H4*: FC positively impacts students’ continued intention to use ELP for college English study.

*Hedonic motivation (HM)* refers to the perceived happiness and enjoyment that users experience while utilizing a particular technology. According to the experience economy theory, offering unique services and experiences with enjoyment and fun is crucial for winning customers’ hearts (Pine and Gilmore, 2011). Gupta and Dogra (2017) and Coves-Martínez et al. (2023) have both confirmed that the higher level of perceived enjoyment from a new technology can increase the continuous use of the technology. When students become interested in using ELP to learn college English, it can stimulate an internal driving force that encourages them to use the platform for their English studies in the future. Therefore, it can be hypothesized that the higher the level of HM that students experience while using ELP to learn college English, the more likely they are to continue using it for their language studies in the future.

*H5*: HM positively impacts students’ continued intention to use ELP for college English study.

*Learning value (LV)* is used to replace price value in this research. Price value represents the trade-off between the benefits and sacrifices of using a new technology. When using a new information technology, user often weighs the perceived benefits against the costs associated with its use (Venkatesh et al., 2012). From a consumer’s perspective, a product has value if it offers some benefits, while from a learning perspective, learning value is defined as the cognitive trade-off students make between the perceived value of using a new technology for study and the time and effort taken for using it (Ain et al., 2016). While students do not typically have to pay to use new technology, they do have to invest their time and energy in order to gain knowledge and learning value (Ain et al., 2016). It is important for any new technology to offer significant benefits that make the investment of time and energy worthwhile. When the students perceive that the investment of time and energy leads to significant improvements in learning outcomes, they are more likely to invest more time and effort into using it, leading to increased continuous intention (Prasetyo et al., 2021; Zacharis and Nikolopoulou, 2022). In this research, if the use of ELP can improve students’ language skills and proficiency, they are inclined to continue using it for college English study. Therefore, the hypothesis can be made as follows:

*H6*: LV positively impacts students’ continued intention to use ELP for college English study.

*Habit (HB)* refers to individuals’ automatic or habitual use of new technologies. This behavioral performance is cultivated unconsciously and automatically based on experience gained from a series of previous behaviors (Venkatesh et al., 2012). Moreover, this habitual behavior contributes to the formation of cognitive commitment for specific behavior, which is gradually formed but not easy to change (Murray and Häubl, 2007). After an extended period of time, the automatic behavior will achieve a relatively stable and continuous state (Venkatesh et al., 2012). This means that a person’s habitual use of technology will become a consistent and regular behavior that is difficult to change. In the learning context, with regular and repeated use of ELP for course participation, forum discussion, assignment submission, examination, and grade checking, students will develop a habitual positive behavior unconsciously. Habitual positive behavior, formed through regular and repeated use of ELP, increases students’ intention to use it in the long run (Raman and Thannimalai, 2021; Tandon et al., 2021; Xu et al., 2022). Therefore, it can be hypothesized that:

*H7*: HB positively impacts students’ continued intention to use ELP for college English study.

### The mediating effect of E-satisfaction (ESA)

Originally, e-satisfaction referred exclusively to customers’ satisfaction with their past shopping experience on an electronic commerce company’s platform (Anderson and Srinivasan, 2003). However, the current research employs e-satisfaction to measure the degree of satisfaction that students have towards using ELP for their college English studies. Previous studies indicate that the perceived usefulness (PE), ease of use (EE), social approval and support (SI), accessibility of technical support and necessary resources (FC), users’ perceived trade-off between profits and cost (PV), and perceived enjoyment and pleasure (HM) from using a new technology have a direct effect on users’ satisfaction with their experience of using it (Alalwan, 2020; Siyal et al., 2021; Wu et al., 2022; Mishra et al., 2023). In the context of college English study, with social and technical support from others, the ease of using ELP for English study can not only help students finish English learning tasks more efficiently and improve academic performance (LV) with little effort (EE), but also increase their feelings of happiness and pleasure. This would positively influence their satisfaction with using ELP for college English study. Based on the above information, PE, EE, SI, FC, LV and HM can increase students’ e-satisfaction with using ELP for college English study. If ELP can offer real benefits to students and match their expectations, they are inclined to have high e-satisfaction with using it for college English study. Accordingly, they are more motivated to continue using it for college English study in the future(Kim et al., 2019; Alalwan, 2020; Wu et al., 2022; Maduku and Thusi, 2023; Perez-Aranda et al., 2023). Therefore, the following hypotheses can be posited.

*H8*: ESA mediates the relationship between (a) PE, (b) EE, (c) SI, (d) FC, (e) LV, (f) HM and students’ continued intention to use ELP for college English study.

### The mediating effect of habit

E-satisfaction refers to the user’s evaluation of an information system in terms of whether it can reach their needs and expectations (Zeithaml and Bitner, 2003). Research shows that users’ e-satisfaction contributes to habit development and long-term engagement (Tran and Trang, 2018). Favorable user experience plays a key role in developing use habits (Thadani and Cheung, 2011; Wang et al., 2013). Once a habit is formed, users become inclined to automatically and habitually repeat it, making it even more difficult to suppress the habit (Aarts and Dijksterhuis, 2000). The stronger the habit, the more determined the user is to keep using the new technology (Baudier et al., 2018; Gu et al., 2019). If users are satisfied with their early experience of using a new technology, they are more inclined to develop automatic and spontaneous behavior towards using it, making them more likely to continue using it in the future (Amoroso and Lim, 2017; Alalwan, 2020). In the context of learning, if ELP can offer real benefits to students and meet their expectations, they are more likely to have high e-satisfaction towards using it for college English study. With accumulated learning experience, habit could be developed, which naturally increases their continued intention to use it for college English learning in future. Thus, It can be posited as:

*H9*: HB mediates the relationship between students’ e-satisfaction and their continued intention to use ELP for college English study.

## Research design and methodology

### Participants

ELP has become an indispensable part of Chinese higher education, enabling students to continue their studies effectively. This research focuses on undergraduate students, as they are significant in the Chinese higher education system. They were learning college English as part of educational programs with ELP at different public universities across Guangxi. As this study explores the factors that impact students’ continued intention to use ELP for college English study without revealing any specific personal information or human performance, there is no need for ethical agreement. With the help of college English teachers, a participant information form was presented to all participants to ensure transparency and informed participation during class interval. Following this, a participant consent form was provided to get participants’ consent. Only after that, can participants start to answer the questionnaire by scanning the QR code. The students participate voluntarily in this survey and their confidentiality was ensured. They are encouraged to answer the questions according to their real using experience and thoughts about application of ELP for college English study, and all survey data will be used only for research aims.

### Instrument development

This research aims to identify the factors that affect students to continue using the ELP for college English study. To achieve this goal, a quantitative approach is adopted, and data is collected online using an online questionnaire. It includes three parts: the first part explains the nature and purpose of the research and includes a filtering question to confirm whether students have used ELP for college English study; the second part is for demographic information, including gender, age, discipline or majors, year currently studying, number of years’ experience in using ELP; and the third part contains the measurement items for all constructs in this research. The constructs of UTAUT2 were adapted from Ain et al. (2016) and Venkatesh et al. (2012), while the items for e-satisfaction were adapted from Alalwan (2020) and Wu et al. (2022) and those for continued intention from Dağhan and Akkoyunlu (2016). All items were modified to better fit this study. To ensure that the measured items accurately reflect the concept of each construct, this study invited information technology personnel and college English teaching experts to evaluate the face and content validity of the questionnaire. Back-translation was done for all questions to maintain the equivalence and consistency of the questionnaire in different languages (English and Chinese). After pilot testing with a group of students and some English teachers, the questionnaire has improved its clarity and quality (see Table 1). A 5-point scale measured the items, with 1 indicating strong disagreement and 5 indicating strong agreement.

**Table 1:** Measurement items.

Construct	Measurement items	Source
Performance expectancy (PE)	I find e-learning platform(ELP) useful for my English studies.	Venkatesh et al. (2012) and Ain et al. (2016)
	ELP allows me to accomplish English class activities more quickly.	
	ELP increases my English learning productivity.	
Effort expectancy (EE)	ELP is easy to use.	
	Learning how to use ELP is easy for me.	
	My interaction with ELP is clear and understandable.	
Social influence (SI)	My peers who influence my behavior think that I should use ELP.	
	My friends who are important to me think that I should use ELP.	
	My instructors whose opinions that I value prefer that I should use ELP.	
Facilitating conditions (FC)	I have resources to use ELP.	
	I have knowledge to use ELP.	
	A specific person (or group) is available to assist when difficulties arise with ELP.	
Learning value (LV)	ELP is worth more than the time and effort given to it.	
	In less time, ELP allows me to quickly and easily share knowledge with others.	
	ELP gives me the opportunity to decide about the pace of my own learning.	
	ELP gives me opportunity to increase my knowledge and control my success.	
Hedonic motivation (HM)	I feel fun using ELP to learn college English.	
	I enjoy using ELP to learn college English.	
	Using ELP to learn college English is very entertaining.	
Habit (HB)	Using ELP to learn English has become a habit for me.	
	I am addicted to using ELP to accomplish my English study tasks.	
	I must use ELP for my English studies.	
Continued intention (CI)	I intend to continue using ELP for English study in the future.	Dağhan and Akkoyunlu (2016)
	I will continue using ELP for English study in the future.	
	I will regularly use ELP for English study in the future.	
E-satisfaction (ESA)	I am normally willing to adopt ELP for English study.	Alalwan (2020) and Wu et al. (2022)
	I am extremely pleased with ELP for English study.	
	I am joyful to adopt ELP for English study.	
	I am pleased with the way that ELP has carried out for English study.	

### Data collection

Data is collected online through Tencent Questionnaire, a survey platform that allows for the creation of customized questionnaires at a relatively lower cost. Eight hundred and sixty-five respondents completed the questionnaire, answers with consistently neutral responses, diagonal lining responses, and alternating extreme pole responses were deleted. This resulted in 626 valid questionnaires (response rate: 72.4%), including 294 males (47%) and 332 females (53%). The sample had a considerable representation of students from different levels of education; 34.5% were freshmen, 39.1% were sophomores, 20.4% were juniors, and 5.9% were seniors. The students were between the ages of 19 and 23 years old. When it comes to online learning experience, most of the students had 2 to 3 years’ experience in using ELP. Majors covers engineering, art, history, law, economics, management, education, philosophy, literature, and so on. According to the recommended ‘10 times rule’(Wu, 2010), which suggests that the sample size should be ten times the number of indicators, 626 samples were sufficient to proceed further with data analysis.

### Data analysis

**Figure 1:**
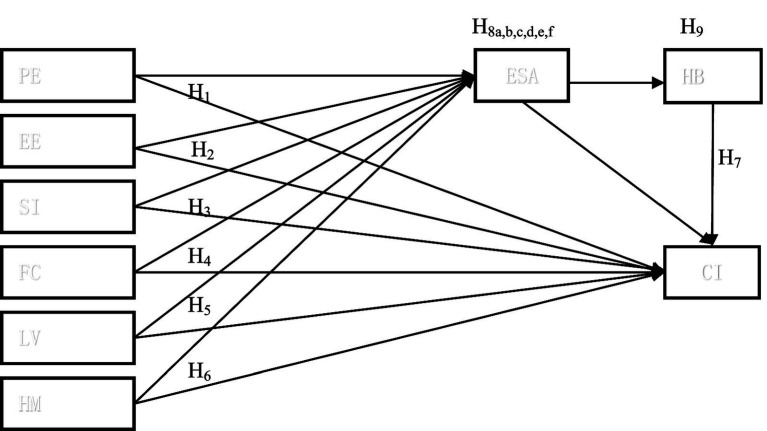
Theoretical framework.

This study uses UTAUT2 to identity the factors that impact students’ continued intention to use ELP for college English study and the mediating effect of e-satisfaction and habit. The proposed model, as shown in Figure 1, displays the variable relationships. The partial least square structural equation modelling (PLS-SEM) was used for data analysis. PLS-SEM is a powerful analytical approach to examine structural equation modeling as it is good at analyzing both indirect and direct effects of the mediating effect (Hair et al., 2017) and it can handle complex models with many structural model relations (Shiau et al., 2019). PLS-SEM model assessment focuses on the evaluation of measurement model and structural model. The former describes the relationships between constructs and their indicators, and the latter deals with the relationships between different constructs and how they interact with each other. In this research, data analysis was conducted in three steps with SmartPLS 3.3: evaluation of measurement model, evaluation of structural model, and test of mediating effect.

## Research results

### Evaluation of measurement model

The evaluation of the measurement model examines the reliability, convergent validity, and discriminant validity of each construct. As shown in Table 2, all Cronbach’s alpha values (0.748 ~ 0.924) and composite reliability values (0.855 ~ 0.952) are higher than 0.7, indicating sufficient internal consistency reliability for all constructs. The factor loading of all constructs (0.784 ~ 0.941) is also higher than 0.7, indicating that the measurement items fully captures the concept of each construct and there is acceptable reliability for each construct. Additionally, the average variance extracted (AVE) of all constructs (0.645 ~ 0.869) is higher than 0.5, indicating good convergent validity for all constructs.

**Table 2:** Reliability and validity results of constructs.

Constructs	Items	Loading	CA	CR	AVE
Performance expectancy (PE)	PE1	0.903	0.893	0.933	0.823
	PE2	0.913			
	PE3	0.907			
Effort expectancy (EE)	EE1	0.875	0.837	0.902	0.753
	EE2	0.858			
	EE3	0.871			
Social influence (SI)	SI1	0.794	0.748	0.855	0.663
	SI2	0.798			
	SI3	0.849			
Facilitating conditions (FC)	FC1	0.860	0.824	0.895	0.740
	FC2	0.877			
	FC3	0.843			
Learning value (LV)	LV1	0.805	0.817	0.879	0.645
	LV2	0.784			
	LV3	0.816			
	LV4	0.806			
Hedonic motivation (HM)	HM1	0.913	0.907	0.942	0.843
	HM2	0.917			
	HM3	0.925			
Habit (HB)	HB1	0.907	0.882	0.927	0.809
	HB2	0.883			
	HB3	0.907			
E-satisfaction (ESA)	ESA1	0.809	0.847	0.896	0.683
	ESA2	0.829			
	ESA3	0.845			
	ESA4	0.823			
Continued intention (CI)	CI1	0.932	0.924	0.952	0.869
	CI2	0.923			
	CI3	0.941			

CA, Cronbach’s alpha; CR, composite reliability; AVE, average variance extracted.

The Fornell-Larcker criterion and heterotrait-monotrait ratio (HTMT) can be used to assess discriminant validity of the constructs (Hair et al., 2017). As shown in Table 3, the diagonal values represent the square root of the AVE of each construct, and each construct’s value is greater than its correlation with other constructs in any column or row, indicating good discriminant validity between the constructs of the measurement model. HTMT assesses the correlation between constructs by comparing the mean of all correlations for measurement items between different constructs and that within the same construct (Henseler et al., 2015). As shown in Table 4, all HTMT correlation values were lower than 0.85, and the 95% confidence intervals do not include the value 1 after bootstrapping, both indicating that there is good discriminant validity for all constructs in the measurement model.

**Table 3:** Discriminant validity with Fornell-Larcker criterion.

	Continued intention	Effort expectancy	Facilitating conditions	Habit	Hedonic motivation	Learning value	Performance expectancy	E-satisfaction	Social influence
Continued intention	0.932								
Effort expectancy	0.518	0.868							
Facilitating conditions	0.525	0.609	0.86						
Habit	0.679	0.47	0.506	0.899					
Hedonic motivation	0.694	0.531	0.561	0.706	0.918				
Learning value	0.693	0.607	0.639	0.655	0.725	0.803			
Performance expectancy	0.617	0.603	0.524	0.568	0.619	0.685	0.907		
E-satisfaction	0.758	0.575	0.575	0.681	0.719	0.699	0.606	0.827	
Social influence	0.322	0.372	0.323	0.318	0.343	0.399	0.522	0.291	0.814

**Table 4:** Discriminant validity with HTMT.

	Continued intention	Effort expectancy	Facilitating conditions	Habit	Hedonic motivation	Learning value	Performance expectancy	E-satisfaction
Effort expectancy	0.584 CI95% [0.505, 0.658]							
Facilitating conditions	0.601 CI95% [0.517, 0.678]	0.733 CI95% [0.673, 0.790]						
Habit	0.749 CI95% [0.688, 0.804]	0.543 CI95% [0.465, 0.613]	0.591 CI95% [0.511, 0.664]					
Hedonic motivation	0.757 CI95% [0.704, 0.805]	0.606 CI95% [0.530, 0.674]	0.648 CI95% [0.572, 0.718]	0.788 CI95% [0.737, 0.834]				
Learning value	0.792 CI95% [0.729, 0.847]	0.732 CI95% [0.655, 0.801]	0.771 CI95% [0.695, 0.839]	0.764 CI95% [0.708, 0.815]	0.837 CI95% [0.791, 0.879]			
Performance expectancy	0.679 CI95% [0.612, 0.741]	0.691 CI95% [0.616, 0.758]	0.61 CI95% [0.527, 0.685]	0.638 CI95% [0.573, 0.695]	0.687 CI95% [0.628, 0.740]	0.805 CI95% [0.752, 0.856]		
E-satisfaction	0.844 CI95% [0.798, 0.885]	0.678 CI95% [0.608, 0.741]	0.685 CI95% [0.613, 0.751]	0.777 CI95% [0.716, 0.831]	0.811 CI95% [0.758, 0.861]	0.824 CI95% [0.763, 0.878]	0.686 CI95% [0.621, 0.744]	
Social influence	0.382 CI95% [0.288, 0.469]	0.466 CI95% [0.379, 0.551]	0.408 CI95% [0.308, 0.502]	0.387 CI95% [0.298, 0.469]	0.412 CI95% [0.323, 0.494]	0.513 CI95% [0.423, 0.596]	0.637 CI95% [0.552, 0.710]	0.348 CI95% [0.255, 0437]

After evaluating the measurement model, the constructs demonstrated satisfactory levels of quality. Prior to evaluating the structural model, variance inflation factor (VIF) was examined to check for collinearity issue. As can be seen in Table 5, all VIF values were below 3.3 (Diamantopoulos and Siguaw, 2006). Therefore, There is no collinearity problem for the structural model in this research.

**Table 5:** Results of *R*^2^, *Q*^2^ and VIF.

Construct	*R^2^*	*Q^2^*	VIF							
			PE	EE	SI	FC	LV	HM	HB	ESA
CI	0.662	0.569	2.527	2.047	1.404	2.009	3.181	2.948	2.385	2.788
ESA	0.616	0.410	2.471	1.999	1.388	1.983	2.989	2.299		
HB	0.464	0.371								1.000

### Evaluation of structural model

In order to predict the variance in the dependent variables, PLS-SEM assessment of the structural model evaluates *R*^2^, *Q*^2^, *f*^2^, and the size and statistical significance of the structural path coefficients (Hair et al., 2017). As shown in Table 5, the *R*^2^ value for e-satisfaction, habit, and continued intention is 0.616, 0.464, and 0.662, respectively. This implies that antecedents can substantially explain the variance in students’ e-satisfaction and their continued intention to use ELM for college English study (Chin, 1998). *Q*^2^ value for e-satisfaction, habit, and continued intention is 0.410, 0.371 and 0.569, respectively, all are larger than zero, indicating that the independent variables have predictive relevance for these dependent variables. Based on the ƒ^2^ values from Table 6, e-satisfaction(ƒ^2^ = 0.156) exerts the largest impact on college students’ continued willingness to use ELM for college English study, and hedonic motivation(ƒ^2^ = 0.162) has the largest effect on students’ e-satisfaction with the application of ELM for college English study. To effectively evaluate the structural model, bootstrapping with 5,000 subsamples is set with two tails.

**Table 6:** Direct relationship results and structural model results.

H	Path	Coef.	*t* value	*p* value	95% Bias-corrected IC	*f* ^2^	Result
H_1_	PE → CI	0.115	2.742	0.006	[0.031, 0.198]	0.016	Yes
H_2_	EE → CI	−0.006	0.154	0.878	[−0.086, 0.071]	0.000	No
H_3_	SI → CI	−0.004	0.151	0.880	[−0.056, 0.048]	0.000	No
H_4_	FC → CI	−0.004	0.096	0.923	[−0.077, 0.073]	0.000	No
H_5_	LV → CI	0.152	3.176	0.002	[0.057, 0.243]	0.022	Yes
H_6_	HM → CI	0.123	2.627	0.009	[0.036, 0.221]	0.015	Yes
H_7_	HB → CI	0.172	3.953	0.000	[0.083, 0.252]	0.037	Yes
	PE → ESA	0.127	3.029	0.002	[0.047, 0.209]	0.017	
	EE → ESA	0.129	3.613	0.000	[0.059, 0.199]	0.022	
	SI → ESA	−0.075	2.354	0.019	[−0.139, −0.014]	0.010	
	FC → ESA	0.096	2.416	0.016	[0.022, 0.179]	0.012	
	LV → ESA	0.227	4.551	0.000	[0.123, 0.321]	0.045	
	HM → ESA	0.378	8.419	0.000	[0.288, 0.462]	0.162	
	ESA → CI	0.383	6.437	0.000	[0.273, 0.505]	0.156	
	ESA → HB	0.681	25.651	0.000	[0.622, 0.728]	0.867	

^*^p < 0.05; ** < 0.01; ***p < 0.001 (two-tails).

From the path coefficients in Table 6, it is evident that PE, LV, HM, and HB have a positive impact on students’ continuous intention to use ELM for college English study, with HB showing the strongest influence, followed by LV, HM, and PE. All *t*-values are >1.96, all *p*-values are <0.05, and their 95% confidence intervals do not contain the value zero, indicating that all these relationships are significant. Therefore, PE, LV, HM, and HB have a significant and positive impact on students’ willingness to continue using ELM for college English study, and hence, *H1*, *H5*, *H6*, and *H7* are supported. On the other hand, EE, SI, and FC do not have a significant effect on students’ willingness to continue using ELM for college English study. In the path from EE to CI, the *t*-value (0.154) is <1.96, the *p*-value (0.878) is above 0.05, and the 95% confidence intervals [−0.086, 0.071] contains the value zero, indicating that EE does not have a significant effect on CI, and therefor, *H2* is not supported. In the path from SI to CI, the *t*-value (0.151) is <1.96, the *p*-value (0.880) is >0.05, and the 95% confidence interval [−0.056, 0.048] contains the value zero, indicating that SI does not have a significant effect on CI, and therefore, *H3* is not supported. In the path from FC to CI, the t-value (0.096) is <1.96, the *p*-value (0.923) is >0.05, and the 95% confidence interval [−0.077, 0.073] contains the value zero, indicating that FC does not significantly impact CI, and therefore, *H4* is not supported.

### Mediating effect analysis

This research follows Zhao et al. (2010) procedures for mediation analysis, using 5,000 bootstrapping samples. A mediation analysis was conducted to assess the impact of e-satisfaction on the relationship between PE, EE, SI, FC, LV, HM, and CI, as well as the effect of habit on the relationship between e-satisfaction and CI. All results are displayed in Table 7.

**Table 7:** Mediating analysis.

H	Path	Total indirect effects/P1xP2				Direct effects/P3				Mediation type	Result
		Coef.	*t* value	*p* value	95% Bias-corrected IC	Coef.	*t* value	*p* value	95% bias-corrected IC		
H8a	PE → ESA → CI	0.049	2.815	0.005	[0.019, 0.088]	0.115	2.742	0.006	[0.031, 0.198]	Complementary partial mediation	Yes
H8b	EE → ESA → CI	0.05	3.243	0.001	[0.023, 0.084]	−0.006	0.154	0.878	[−0.086, 0.071]	Full mediation	Yes
H8c	SI → ESA → CI	−0.029	2.091	0.037	[−0.061, −0.006]	−0.004	0.151	0.88	[−0.056, 0.048]	Full mediation	Yes
H8d	FC → ESA → CI	0.037	2.426	0.015	[0.010, 0.071]	−0.004	0.096	0.923	[−0.077, 0.073]	Full mediation	Yes
H8e	LV → ESA → CI	0.087	3.800	0.000	[0.048, 0.139]	0.152	3.176	0.002	[0.057, 0.243]	Complementary partial mediation	Yes
H8f	HM → ESA → CI	0.145	4.648	0.000	[0.093, 0.216]	0.123	2.627	0.009	[0.036, 0.221]	Complementary partial mediation	Yes
H9	ESA → HB → CI	0.117	3.845	0.000	[0.056, 0.175]	0.383	6.437	0.000	[0.273, 0.505]	Complementary partial mediation	Yes

Regarding the three mediating paths of *PE → ESA → CI, LV → ESA → CI*, and *HM → ESA → CI*, the results indicate that their indirect and direct effects are significant. Additionally, all 95% confidence intervals of both indirect and direct effects exclude zero, indicating that e-satisfaction positively and partially mediates these relationships. To determine the type of partial mediation, the product of the direct effect (positive) and the indirect effect (positive) is positive, which supports the idea that e-satisfaction represents complementary partial mediation for these three paths. These findings support *H8*a, *H8*e, and *H8*f.

In the paths *EE → ESA → CI, SI → ESA → CI*, and *FC → ESA → CI*, all indirect effects are significant while direct effects are insignificant. Furthermore, the 95% confidence intervals for the indirect effects exclude zero, while the direct effects include zero. Therefore, e-satisfaction fully mediates the relationships along these three paths. As a result, *H8*b, *H8*c, and *H8*d are supported.

In the path *ESA → HB → CI,* the 95% confidence intervals for both the indirect effect [0.056, 0.175] and the direct effect [0.273, 0.505] exclude zero. This indicates that *habit* partially mediates this relationship, since the indirect and direct effects are significant. The fact that the product of the direct effect (positive) and the indirect effect (positive) is positive indicates that habit reveals complementary partial mediation for this path, which supports *H9*.

## Discussion

Based on the extended UTAUT2, this research has conducted a thorough analysis of the factors that impact students’ e-satisfaction and willingness to continue using ELP for college English study. In addition to identifying these determinants, the study has also tested the mediating role of e-satisfaction and habit. The model has high explanatory power for the variance of students’ e-satisfaction and continued intention to use ELP for college English study. The research results show that PE, LV, HM, and HB significantly affects students’ willingness to continue using ELP to learn college English, while EE, SI, and FC have no significant impact. E-satisfaction mediates the relationship between PE, EE, SI, FC, LV, HM, and CI, while habit positively mediates the relationship between e-satisfaction and CI.

More specifically, PE significantly influences students’ intention to continue using ELP for college English study. This finding is in confirmation with the results of previous research by Chen et al. (2018), Mishra et al. (2023), and Wu et al. (2022), which held that the cognitive and functional usefulness are important factors for saving time and effort. The more efficiently that students perceived they can use ELP to complete class activities and assignments, the higher their intention to continue using it for further study. Therefore, it is crucial for educators to emphasize the importance of using ELP in the classroom to maximize its benefits and encourage its continued use among students. And what’s more, the result provides valid reason to recommend ELP developers to further develop the technologies and improve the functions for students to increase learning efficiency in college English study.

The findings of this study demonstrate that LV positively influences CI. This result corroborates the findings of previous studies, such as those conducted by Ain et al. (2016), Dajani and Abu Hegleh (2019), Prasetyo et al. (2021), and Zacharis and Nikolopoulou (2022), which held that the use of ELP for college English study increases students’ perception of academic value, leading to greater willingness to continue using it in the future. This is particularly important given the increasing importance of English proficiency in the global job market and the need for students to have access to effective language learning tools. By taking advantage of ELP, students can improve their English language skills and gain a competitive edge in their future careers. Additionally, the use of ELP has been shown to have broader benefits beyond simply improving language proficiency, such as enhancing critical thinking skills and increasing cultural awareness. Therefore, it is important for educators to recognize the potential of ELP and incorporate it into teaching strategies to increase students’ participation in college English learning.

The results of the current research suggest that HM exerts a significant and positive impact on CI, which is consistent with previous research results from Alalwan (2020) and Liu et al. (2022), showing that the students are willing to continue using ELP in the future if they derive pleasure and enjoyment from using it during their college English studies. It is worth noting that the use of ELP contributes to a more immersive and stimulating language learning experience, which can further enhance students’ language proficiency. The results of this research can have important implications for educators who are trying to find effective ways to enhance students’ motivation and engagement for better learning outcomes.

The research results indicate that HB was the most significant factor in predicting CI to use ELP for college English study. This finding confirms with previous studies by Veeramootoo et al. (2018), Gu et al. (2019), Tandon et al. (2021), and Xu et al. (2022) which have also demonstrated the substantial impact of HB on CI. The importance of fostering positive habitual behavior towards the use of ELP for college English study cannot be overstated. By developing such positive habits, students will be more motivated to use ELP for college English study in the future. Additionally, it might be worthwhile to investigate ways to encourage the development of such habits and to identify factors that might hinder their development. Therefore, it is crucial for universities to offer support and adequate resources to help students develop positive attitudes and create a supportive learning environment that encourages ELP use in college English study.

However, the empirical results of this research failed to confirm the role of EE, SI, and FC in predicting CI. It is worth noting that there may be several reasons for this. With regard to EE, one possible reason is that students have attached more importance to the usefulness of ELP (Prasetyo et al., 2021). In other words, if students perceive a high level of value and benefits, they can overcome any difficulties in using this technology (Davis et al., 1992). Another reason is that students are proficient in using various new information technologies, and ELP is not a complex system for completing English class activities and tasks. This suggests that students may have found ELP to be quite useful and easy to use, which could explain the lack of significance in the relationship between EE and CI. Regarding SI, one reason may be that ELP is the best way to continue studying during the pandemic period, and students can realize the importance of taking responsibility for their own learning without the influence of peers, friends, teachers, and classmates (Tandon et al., 2021). This self-motivated approach could explain why the impact of social influence on ELP adoption is not as significant as initially hypothesized. It is also worth noting that the impact of social influence may diminish over time, as students gain more experience with using new information technologies (Alalwan, 2020). However, it is important to consider that some students may still require social support and encouragement to adopt ELP. Therefore, it is important to provide a supportive learning environment that encourages students to use ELP for college English study. With regard to FC, it is true that ELP is quite simple from the perspective of knowledge dissemination (Xu et al., 2022). Moreover, with adequate experience using different new technologies, students are less likely to be affected by FC to keep using it continuously (Venkatesh et al., 2012). This suggests that FC may be less important when students have sufficient experience using similar technologies, which could explain the lack of significance in the relationship between FC and IC. However, it is important to consider that some students may require additional resources and support to ensure that they are able to continue using ELP for college English study, for example, providing training sessions and technical support can help students overcome any difficulties they may encounter.

The partial mediating effect of e-satisfaction on the relationship between PE, LV, HM, HB, and CI implies that these relationships are not direct causal ones. Instead, PE, LV, HM, and HB influence e-satisfaction, which in turn influences CI. The fact that the partial mediation of e-satisfaction implies that it explains some, but not all of these relationships. This raises the possibility that other potential mediating variables, which were not examined in this study, may further clarify the nature of these relationships. In contrast, e-satisfaction fully accounts for the relationship between EE, SI, FC, and CI, as shown by the full mediating effect of e-satisfaction. This finding supports the hypothesized theoretical framework for these relationships. The partial mediating effect of habit on the relationship between e-satisfaction and CI implies that the relationship is not a direct causal one. Rather, e-satisfaction influences habit, which in turn influences CI. The partial mediation of habit implies that it explains some, but not all for this relationship. Other potential mediating variables may further clarify the nature of this relationship.

## Conclusion

This research, based on the extended UTAUT2, explores the determinants for continued intention to use ELP and tests the mediating effect of e-satisfaction on the relationships between antecedents and CI, as well as the mediating effect of habit on the relationship between e-satisfaction and CI. The model has high explanatory and predicative power in this research. Results show that PE, LV, HM, and HB significantly affect students’ continued intention to use ELP to learn college English. E-satisfaction and habit play a mediating role in the proposed relationships. This study is helpful for education administrators, particularly online education policy makers, to better understand the determinants that would increase students’ learning efficiency, participation and satisfaction with using ELP for college English study. The research results can guide online education policy makers and ELP designers to increase the effectiveness of ELP and improve students’ usage experience, leading to greater satisfaction, higher learning efficiency and participation with continued use of ELP in future college English study.

Theoretically, this research aimed to broaden the scope and theoretical depth of UTAUT2 by examining the role of e-satisfaction and habit as mediating factors, rather than simply investigating the determinants of students’ continued intention to use ELP for college English study. To achieve this, this research introduces e-satisfaction and learning value as additional components of the UTAUT2 framework in the context of college English study. The results show that e-satisfaction not only significantly affect students’ willingness to continue using new technology, but also plays a partial and full mediating effect in the relationship between antecedents and CI. Furthermore, the research confirms the mediating effect of habit on the relationship between e-satisfaction and CI. By identifying the mediating effect of e-satisfaction and habit, this study contributes to a better understanding of the factors that would increase students’ learning efficiency, participation, and satisfaction with using ELP, which can also increase the successful implementation of ELP for college English study.

Practically, this research provides suggestions for online education policy makers to increase students’ learning efficiency, participation and satisfaction with using ELP for college English study. In order to increase students’ learning efficiency, universities must take steps to raise students’ awareness of ELP’s usefulness and functions. This can be accomplished by emphasizing how ELP can help students complete class activities and tasks more efficiently. Students’ participation can be increased by enhancing the usability of ELP. Designer should consider simplifying its operation procedures and categorizing learning resources, which would help students operate the platform more easily and efficiently, saving them time and energy. And what’s more, it would be better for designers to integrate a game incentive mechanism into ELP. Gamifying the learning activities can offer more happiness and pleasure to students, making them more likely to participate the class activities. By using game incentive mechanisms, the learning experience can be made more exciting and interesting for the students. If students are satisfied with the learning experience, they are more likely to repeat the behavior. Once habitual behavior is developed, students will continuously use ELP for their daily study.

Although the research findings provide new insights for the successful implementation of ELP for college English study, they still have some limitations. Firstly, this cross-sectional research only reflects the students’ perception and attitude towards using ELP for college English study at a certain point in time. Conducting longitudinal research could tract students’ perception and experience with using a new technology over time. Secondly, the study has only examined the mediating effect of e-satisfaction and habit within the UTAUT2 model. There might be other potential mediators or moderators that could further explain the relationship between the antecedents and the continued willingness to use new technologies in the context of education. Thirdly, in the context of foreign language learning with information technology, students’ language proficiency and interaction with instructors and peers have a great impact on their willingness to continuously use a certain new technology (Deng et al., 2019). Further research could incorporate language competence and interactive quality into the model to study its impact on students’ satisfaction and continued intention to use ELP for foreign language study. Additionally, the research has not considered the influence of students’ engagement and self-regulation on their continued intention to use ELP.

## Data availability statement

The raw data supporting the conclusions of this article will be made available by the authors, without undue reservation.

## Author contributions

All authors listed have made a substantial, direct, and intellectual contribution to the work and approved it for publication.

## Funding

This study was supported by the Department of Education of Guangxi, China (Grant No. 2022JGB364).

## Conflict of interest

The authors declare that the research was conducted in the absence of any commercial or financial relationships that could be construed as a potential conflict of interest.

## Publisher’s note

All claims expressed in this article are solely those of the authors and do not necessarily represent those of their affiliated organizations, or those of the publisher, the editors and the reviewers. Any product that may be evaluated in this article, or claim that may be made by its manufacturer, is not guaranteed or endorsed by the publisher.
